# Evidence-based recruitment strategies for clinical research: Study personnel’s and research participants’ perceptions about successful methods of outreach for a U.S. Autism-Research Cohort

**DOI:** 10.1017/cts.2024.512

**Published:** 2024-04-02

**Authors:** Robin P. Goin-Kochel, Ivana Lozano, Gabrielle Duhon, Gabriela Marzano, Amy Daniels, J. Kiely Law, Katharine Diehl, LeeAnne Green Snyder, Pamela Feliciano, Wendy K. Chung, SPARK Consortium

**Affiliations:** 1 Baylor College of Medicine, Houston, TX, USA; 2 Texas Children’s Hospital, Houston, TX, USA; 3 Simons Foundation, New York, NY, USA; 4 Boston Children’s Hospital, Harvard Medical School, Boston, MA, USA

**Keywords:** Research recruitment, participant enrollment, autism, parent/caregiver

## Abstract

**Introduction:**

Under enrollment of participants in clinical research is costly and delays study completion to impact public health. Given that research personnel make decisions about which strategies to pursue and participants are the recipients of these efforts, we surveyed research staff (*n* = 52) and participants (*n* = 4,144) affiliated with SPARK (Simons Foundation Powering Autism for Knowledge) – the largest study of autism in the U.S. – to understand their perceptions of effective recruitment strategies.

**Methods:**

In Study 1, research personnel were asked to report recruitment strategies that they tried for SPARK and to indicate which ones they would and would not repeat/recommend. In Study 2, SPARK participants were asked to indicate all the ways they heard about the study prior to enrollment and which one was most influential in their decisions to enroll.

**Results:**

Staff rated *speaking with a SPARK-study-team member (36.5%), speaking with a medical provider (19.2%), word of mouth (11.5%), and a live TV news story (11.5%) as the most successful strategies.* Participants most often heard about SPARK via *social media (47.0%), speaking with a medical provider (23.1%), and an online search (20.1%). Research personnel’s and participants’ views on effective recruitment strategies often differed, with the exception of speaking with a medical provider.*

**Conclusion:**

Results suggest that a combination of strategies is likely to be most effective in reaching diverse audiences. Findings have implications for the selection of strategies that meet a study’s specific needs, as well as recruitment-strategy “combinations” that may enhance the influence of outreach efforts.

## Introduction

Recruitment for clinical studies remains one of the biggest challenges to performing studies of all sizes. As many as 75% of studies are unable to enroll the proposed number of participants and 90% fail to enroll within the original timeframe [1]. Study preparation and startup costs comprise the largest proportion of expenses in clinical research [2], and extensions granted to low-enrolling studies further increase costs and delay results that could have important public-health implications. Unfortunately, many studies are eventually abandoned as a result of failing to meet enrollment goals. A report from Oregon Health and Science University revealed that the university spent nearly $1 million in fiscal year 2009 on studies that terminated early because of low enrollment numbers [2]. Not only do these investments fail to produce any scientific results [3] but they also indirectly prevent other research studies from being conducted that could produce results. For these reasons, research dollars need to be allocated to recruitment activities that yield the greatest return on investment. Understanding which recruitment methods are most likely to successfully enroll participants is thus of significant importance financially, scientifically, and ethically.

### Common recruitment challenges

Research teams experience a range of challenges that contribute to low enrollment in clinical studies. Factors like the topic of study, invasiveness or riskiness of procedures, duration of participation, and perceived cost/benefit ratio may all influence a potential participant’s willingness to participate. For example, during a survey of individuals aged 14–21 with post-traumatic stress disorder who were participating in a clinical trial, approximately 60% of respondents reported the demands of the study to be a significant barrier to participation, including video recordings, problems with transportation, and diagnostic procedures [4]. In studies that rely heavily on referrals from healthcare providers, gatekeeping—or the reluctance to share research opportunities with potentially eligible patients—can hinder recruitment efforts and lead to selection bias [5]. Relatedly, ensuring racial and ethnic representativeness can be difficult, as research has identified several barriers to participation among communities of color. In particular, Black/African American individuals commonly cite concerns about racial discrimination and mistrust of healthcare/research staff as barriers to participation in genetic research [6-9], while Asian families report language barriers and a lack of information/resources available in their native languages [8]. Fortunately, recent studies leveraging tools within the electronic health record (EHR) for research recruitment demonstrate both (a) improved collaboration between healthcare providers and researchers and (b) increased engagement and retention of families of color in clinical research [10-12]. Efforts like these are critical for identifying effective recruitment strategies that counteract barriers to research participation and expedite study progress.

### Effective recruitment strategies

Several types of recruitment methods have been described as facilitating recruitment and retention efforts for clinical-research studies, with some of the most common being social media advertising, physician referrals, and study flyers [4,13]. In one study that compared the effectiveness of paid social media advertising (i.e., Facebook) with a radio-based recruitment campaign, social media demonstrated superior outreach at a substantially lower cost [14]. Similar work that compared Facebook advertising with traditional recruitment methods (e.g., flyers, newspaper ads, radio, word of mouth) found that, while the former was more cost-effective during initial outreach and screening efforts, it was less cost-effective in terms of actual enrollment and study completion; as such, the authors recommended using a variety of recruitment strategies simultaneously [15]. Other strategies described as effective include sending personalized letters to potentially eligible participants, using telephone reminders throughout the study, and providing financial incentives [16,17]. Additionally, being flexible in terms of when study visits are offered (e.g., including evenings and weekends) and actively engaging clinic staff in the identification of potential participants were successful strategies for recruiting parents in behavioral intervention trials [18]. Furthermore, targeted recruitment of particular racial or ethnic groups should include sociocultural-specific methods [16], such as recruiting through places of worship and having the study team reflect similar racial, ethnic, and language backgrounds of the participants. Collectively, this information provides an evidence base for the range and relative success of various recruitment strategies. However, the majority of this work focuses on adult patient populations with mental and medical illnesses who enrolled in treatment-intervention studies. Specific to recruitment in pediatric research, investigators from one study observed that *type* of recruitment method was key, with in-person recruitment being the only factor significantly associated with achieving 80% of enrollment goals [19]. These results were similar to those of another study that demonstrated active (direct patient outreach) versus passive (flyers, posters, social media, press release) recruitment strategies as superior in engaging families for a pediatric clinical trial addressing obesity, with active strategies effectively recruiting 95% of the total sample [20]. Literature on this topic is sparse, however, and virtually nothing is known about strategies that are most effective for recruitment of pediatric populations with developmental disabilities, where caregivers often decide on behalf of their minor children and/or dependents whether to participate.

Given that (a) investigators and their research teams choose which recruitment strategies to pursue and (b) parents/caregivers are frequently the recipients of these outreach efforts for studies targeting children/dependents, it is valuable to examine these groups’ perceptions about which recruitment methods are most successful in facilitating participant enrollment. Investing time and financial resources in strategies that resonate most with the target audience stands to maximize study enrollment while improving efficiency. Study teams can use this information to evaluate their current recruitment practices and determine whether and where changes may be needed. With this in mind, the purpose of the current study was to assess study personnel’s and research participants’ perspectives about effective recruitment strategies for the national SPARK cohort of individuals with autism and their family members.

### SPARK cohort

As described in *An evaluation of participant recruitment in SPARK, a large, online longitudinal research study of* autism [21], SPARK (Simons Foundation Powering Autism Research for Knowledge) is a national, U.S. study led by the Simons Foundation Autism Research Initiative to identify new genetic contributions for autism and accelerate the pace of autism research [22]. A key goal for SPARK is to sequence the DNA of 50,000 individuals with autism and both biological parents (trios). To reach this goal, SPARK enlisted the help of more than 30 clinical sites across the U.S. to recruit participants and assist them with study completion. The clinical-site network operated under a single independent regulatory board (Western Institutional Review Board – Copernicus Group [WIRB-CG]). As such, all sites had IRB approval to conduct the same research activities; however, site-specific institutional barriers may have prevented some sites from pursuing particular activities. Each recruitment site was granted between $150,000 to $200,000 annually (inclusive of 20% indirect costs) to enroll a certain number of families each year. The budget was allocated at the PIs’ discretion for personnel, supplies, and recruitment/outreach activities. The current assessment specifically focused on participant recruitment at the site level to afford a granular view of these activities relative to the broader outreach efforts of the central SPARK team at the Simons Foundation. For a complete overview of these SPARK central efforts, please see Daniels et al. [21] Consistent with this paper, we refer to the “primary account holder” as the individual in the family who initiated participation in SPARK and “clinical-site affiliation” as the primary account holder’s self-selected association with a clinical site in the SPARK network. Both studies described below were reviewed and approved by the Baylor College of Medicine Internal Review Board.

## Studies: Methods and results

### Study 1: Study teams’ perceptions of effective recruitment strategies

#### Participants

We invited 150 current or past coordinators and principal investigators (PIs) from 25 SPARK sites (active as of June 2018) to participate in a survey about the recruitment strategies that their site had used and which they felt were most successful in influencing families to enroll. A total of 52 individuals responded from 23 sites, for a response rate of 35%; 32 (61.5%) were in a coordinator role, 13 (25.0%) were in a PI role, 5 (9.6%) were in some other role, and 2 (3.8%) were no longer working on the project. In terms of their time/effort allocated to SPARK, 17 (32.7%) contributed 75% effort or more, 10 (19.2%) contributed between 25%–74% effort, 6 (11.5%) contributed between 11%–24% effort, and 19 (36.5%) contributed ≤ 10% effort. Before joining SPARK, 39 (75.0%) had prior experience recruiting for a clinical research study.

#### Procedure

We developed a REDCap online questionnaire that asked about recruitment strategies that study teams had used for SPARK, which strategies they believed were most influential in participants’ decisions to enroll, perceived cost of each strategy, and which strategies they believed were most effective overall. An initial list of 20 specific recruitment strategies was presented in the questionnaire, with three open-ended spaces for participants to provide additional strategies not listed (see Table 1 for a complete list and description provided to participants). Email invitations to complete this questionnaire were distributed to potential participants, with up to three email reminders sent to non-responders. The survey was open for six days prior to a SPARK all-site investigator meeting, at which aggregate results from the survey were shared. Participants were incentivized with a chance to have their names drawn to receive one of five $100 gift cards. Data were analyzed using SPSS v. 28 analytical software.

**Table 1.** Recruitment strategies employed for SPARK and study personnel’s frequency of use

Recruitment Strategy	Description/Examples	Use Frequency^a^ (%)
Print materials	SPARK brochures, post cards, flyers, posters, etc.	52 (100%)
Paid social media	Facebook, Twitter, Instagram sponsored ads	44 (84.6%)
Non-paid social media	Organically sharing/posting on hospital or clinic social media pages, autism-related groups, or via community organizations who share on your behalf	45 (86.5%)
Paid radio advertising	Paid radio campaign/advertising with a prerecorded script that runs for a specified number of weeks at specific times	20 (38.5%)
Radio or podcast interview	Invitation by a radio/podcast host to interview you about SPARK on air/for a prerecorded podcast, at no cost to you	20 (38.5%)
Radio PSA^b^	A script or prerecorded audio file submitted to various radio stations to share if they so choose; PSAs are not guaranteed slots and must be “accepted,” but are free-of-charge because they benefit the community	10 (19.2%)
Mass letter mailing (500+)	Letters mailed to a large number of families/stakeholders inviting them to join SPARK. This may include patient registries, clinic lists, data pulls from the electronic health record, newsletter distribution list, etc.	32 (61.5%)
Medical providers in clinic	Medical providers sharing print materials and information with potentially eligible families during previously scheduled clinic appointments at your site	48 (92.3%)
Blog post	Current participant, study team member, clinical provider, or other stakeholder writes a blog that directly relates to the SPARK study/directs families to a SPARK-related website	13 (25.0%)
Mass email blasts (500+, including newsletters)	Emails sent to a large number of families/stakeholders inviting them to join SPARK. This may include patient registries, clinic lists, data pulls from the electronic health record, newsletter distribution list, etc.	41 (78.8%)
Online news story	Interview/story published online by a news station/group, or a news station sharing a press release about SPARK from your institution	34 (65.4%)
TV news story or interview	Invitation by a TV news or talk-show host to interview you/study personnel about SPARK, to be played on air	35 (67.3%)
TV PSA^b^	Submitting a video file to various TV stations to share freely, if they so choose. PSAs are not guaranteed slots and must be “accepted,” but are free-of-charge because they benefit the community	8 (15.4%)
Paid Google advertising	Paid advertising campaigns for Google that result in your information showing up “first” in search results for specified key words	4 (7.7%)
Paid website advertising	Paying a website to place your information as a banner ad, side-bar ad, etc., for their viewers to see (e.g., news websites, community-partner sites)	11 (21.2%)
Hospital/clinic official website	Listing SPARK on any webpage owned by your institution (e.g., www.bcm.edu, www.texaschildrens.org) This could include a faculty lab page, a larger list of research opportunities at your institution, a specific center’s page, etc.	40 (76.9%)
SPARK coordinators in clinic	SPARK coordinators meeting with families as they come through clinic for previously scheduled appointments, or inviting families into clinic specifically to meet with a SPARK coordinator	43 (82.7%)
Walks, informational events, conferences	Attending local or distant autism-related events/conferences as an exhibitor or speaker to share information about SPARK	49 (94.2%)
On-site registration/saliva-collection events	Inviting families to register/collect saliva in person with the study team as part of an outreach event	45 (86.5%)
Transit advertising	Placing banner ads on city transit systems (e.g., buses, subways, trains)	7 (13.5%)

*Note*: ^a^ *N* = 52; ^b^PSA = Public service announcement.

#### Results

The average number of recruitment strategies that study personnel indicated they had used for SPARK was 11.8 (*SD* = 2.9, range = 4–18). The frequencies and proportions of participants using each type of strategy are shown in Table 1. Other recruitment methods listed in open-ended format that did not fit into a category included advertising prior to previews at movie theaters, smaller email blasts (i.e., < 500), and attending non-autism-specific events (e.g., festivals, state fairs). The methods most frequently endorsed as being most influential in families’ decisions to participate included *speaking with a SPARK team study-team member* (*n* = 19, 36.5%), *speaking with a medical provider* (*n* = 10, 19.2%), *word of mouth* (*n* = 6, 11.5%), and *a live TV news story* (*n* = 6, 11.5%).

In terms of perceived costs of recruitment strategies, participants rated most strategies as “least” or “somewhat” costly, with the highest proportions indicating (a) *unpaid social media* (*n* = 44, 97.8%), *mass email blasts* (*n* = 39, 97.5%), *hospital/clinic website* (*n* = 38, 97.4%), and *blog post* (*n* = 12, 91.7%) as least costly and (b) *transit advertising* (*n* = 6, 100%), *paid radio advertising* (*n* = 12, 63.1%), *on-site registration/saliva-collection events* (*n* = 16, 36.3%) and *paid website advertising* (*n* = 3, 30.0%) as most costly (Table 2). In terms of recruitment methods that study personnel had tried and believed were most effective overall, participants indicated the following strategies that they were likely to repeat/recommend or rate as one of the best: *speaking with SPARK team members in clinic* (*n* = 37, 86.0%), *on-site registration/saliva-collection events* (*n* = 32, 72.7%), *paid social media* (*n* = 26, 59.0%), *speaking with a medical provider in clinic* (*n* = 25, 52.0%), and *printed materials* (*n* = 27, 51.9%). The following were indicated as strategies that they would not repeat/recommend: a *blog post* (*n* = 10, 71.4%), *radio PSA* (*n* = 6, 66.7%), and a *radio or podcast interview* (*n* = 11, 52.4%) (Table 3). Study personnel most often believed that a family heard about SPARK between two and four times before enrolling (*n* = 40, 76.9%).

**Table 2.** Recruitment strategies employed for SPARK and study personnel’s ratings of cost*

Recruitment Strategy	Least or Somewhat Costly *n* (%)	Moderately Costly *n* (%)	More or Most Costly *n* (%)
Print materials (*n* = 51)	23 (45.1%)	21 (41.2%)	7 (13.7%)
Paid social media (*n* = 43)	19 (44.2%)	13 (30.2%)	11 (25.6%)
Non-paid social media (*n* = 45)	44 (97.8%)	1 (2.2%)	0 (0%)
Paid radio advertising (*n* = 19)	2 (10.5%)	5 (26.3%)	12 (63.1%)
Radio or podcast interview (*n* = 19)	15 (78.9%)	2 (10.5%)	2 (10.5%)
Radio PSA^b^ (*n* = 10)	8 (80.0%)	10 (10.0%)	10 (10.0%)
Mass letter mailing (500+) (*n* = 32)	12 (37.5%)	12 (37.5%)	8 (25.0%)
Medical providers in clinic (*n* = 47)	38 (80.8%)	6 (12.8%)	3 (6.4%)
Blog post (*n* = 12)	11 (91.7%)	1 (8.3%)	0 (0%)
Mass email blasts (500+, including newsletters) (*n* = 40)	39 (97.5%)	1 (2.5%)	0 (0%)
Online news story (*n* = 33)	28 (84.8%)	2 (6.1%)	3 (9.1%)
TV news story or interview (*n* = 34)	29 (85.3%)	4 (11.8%)	1 (2.9%)
TV PSA^b^ (*n* = 7)	6 (85.7%)	1 (14.3%)	0 (0%)
Paid Google advertising (*n* = 4)	2 (50.0%)	2 (50.0%)	0 (0%)
Paid website advertising (*n* = 10)	3 (30.0%)	4 (40.0%)	3 (30.0%)
Hospital/clinic official website (*n* = 39)	38 (97.4%)	1 (2.6%)	0 (0%)
SPARK coordinators in clinic (*n* = 42)	28 (66.7%)	9 (21.4%)	5 (11.9%)
Walks, informational events, conferences (*n* = 48)	24 (50.0%)	14 (29.2%)	10 (20.8%)
On-site registration/saliva-collection events (*n* = 44)	14 (31.8%)	14 (31.8%)	16 (36.3%)
Transit advertising (*n* = 6)	0 (0%)	0 (0%)	6 (100%)

*Note*: ^a^ *N* = 52; ^b^PSA = Public service announcement. *Study personnel were asked: “Besides personnel and basic office supplies, how costly is it to execute this recruitment strategy at your site?.”

**Table 3.** Recruitment strategies employed for SPARK and study personnel’s ratings of efficacy

Recruitment Strategy	Unlikely to Repeat/ Recommend *n* (%)	Somewhat Likely to Repeat/ Recommend *n* (%)	Highly Likely to Repeat/ Recommend *n* (%)
Print materials (*n* = 52)	10 (19.2%)	15 (28.9%)	27 (51.9%)
Paid social media (*n* = 35)	9 (20.5%)	9 (20.5%)	26 (59.0%)
Non-paid social media (*n* = 46)	14 (30.4%)	14 (30.4%)	18 (39.1%)
Paid radio advertising (*n* = 20)	9 (45.0%)	8 (40.0%)	3 (15.0%)
Radio or podcast interview (*n* = 21)	11 (52.4%)	7 (33.3%)	3 (14.3%)
Radio PSA^b^ (*n* = 9)	6 (66.7%)	2 (22.2%)	1 (11.1%)
Mass letter mailing (500+) (*n* = 33)	12 (36.4%)	7 (21.2%)	14 (42.4%)
Medical providers in clinic (*n* = 48)	14 (29.2%)	9 (18.8%)	25 (52.0%)
Blog post (*n* = 14)	10 (71.4%)	3 (21.4%)	1 (7.2%)
Mass email blasts (500+, including newsletters) (*n* = 41)	12 (29.3%)	10 (24.4%)	19 (46.3%)
Online news story (*n* = 34)	10 (29.4%)	10 (29.4%)	14 (41.2%)
TV news story or interview (*n* = 35)	8 (22.8%)	12 (34.3%)	15 (42.9%)
TV PSA^b^ (*n* = 8)	3 (37.5%)	2 (25.0%)	3 (37.5%)
Paid Google advertising (*n* = 4)	2 (50.0%)	2 (50.0%)	0 (0%)
Paid website advertising (*n* = 9)	3 (33.3%)	3 (33.3%)	3 (33.3%)
Hospital/clinic official website (*n* = 40)	17 (42.5%)	13 (32.5%)	10 (25.0%)
SPARK coordinators in clinic (*n* = 43)	2 (4.7%)	4 (9.3%)	37 (86.0%)
Walks, informational events, conferences (*n* = 49)	12 (24.5%)	13 (26.5%)	24 (49.0%)
On-site registration/saliva-collection events (*n* = 44)	9 (20.5%)	3 (6.8%)	32 (72.7%)
Transit advertising (*n* = 6)	2 (33.3%)	2 (33.3%)	2 (33.3%)

*Note*: ^a^ *N* = 52; ^b^PSA = Public service announcement.

If a participant had not used a given recruitment strategy, we asked them to indicate the reasons why. Most reported a lack of knowledge about the strategy as a reason (23.0%), followed by lack of money (17.7%), lack of time (17.3%), lack of personnel (12.5%), lack of institutional support/within-site barriers (11.7%), lack of IRB approval (3.3%), or some other reason (14.5%). When asked how easy or difficult it was to recruit for SPARK compared to other studies, 12 (23.1%) said it was easier, 16 (30.8%) said it was harder, and 12 (23.1%) said it was about the same (12 [23.1%] had no response).

### Study 2: SPARK participants’ perceptions of effective recruitment strategies

#### Participants

We invited primary account holders enrolled in SPARK (i.e., individuals who initiated participation on behalf of themselves and/or their families) and who had (a) consented to provide genetic samples and (b) were affiliated with one of the top 16 performing sites or had no particular site affiliation to participate in a survey about how they learned about SPARK and their understanding of participation. A total of 26,997 primary account holders received invitations about the study; 4,144 completed the survey, for a response rate of 15.3% (response rate range across sites = 12%–18%). Among these participants, 3,617 (87.3%) were parents/legally authorized representatives of minor children or adult dependents with autism and 527 (12.7%) were self-reporting, independent adults with autism; 3,627 (87.5%) reported as female; 2,516 (60.7%) were between the ages of 30–44 and 1,164 (28.1%) were between 45–60; 3,670 (88.6%) were White, 251 (6.1%) were Black/African American, 132 (3.2%) were Asian, 154 (3.7%) endorsed some other race; and 436 (10.5%) were Hispanic/Latinx. A total of 3,425 (82.6%) had provided a sample for the genetic component of SPARK (return of the saliva kit for the individual with ASD in the family), and 1,156 (27.9%) were not affiliated with one of the clinical sites.

At the time of enrollment, all SPARK participants were asked how they heard about the study. If a participant was referred by a clinical site and used a site-specific URL to join, the response to this question was set at “clinical site / hospital / university” from a range of responses in a drop-down menu. Therefore, caution should be taken when interpreting these results, as individuals who were referred by a clinical site could have heard about SPARK in several different ways. For participants of this study, responses to, “How did you hear about us?” were as follows: 2,577 (62.4%) clinical site/hospital/university; 938 (22.7%), online; 144 (3.5%), invited by a family member; 121 (2.9%), media announcement; 116 (2.8%), a friend; 88 (2.1%), the Interactive Autism Network; 68 (1.7%), healthcare provider; 51 (1.2%), a community-based organization; and 26 (0.6%), through some other method.

#### Procedure

We collaborated with the SPARK Research Match team to develop and distribute a questionnaire about how families learned about SPARK, methods they deemed most influential in their decisions to enroll, and their understanding of what participation entailed. Potential participants were invited in six batches from October 2, 2019, to November 6, 2019. The survey was open for two months (from October 2, 2019, to December 2, 2019), with up to three reminder emails sent to non-responders. Participants were incentivized with a chance to have their names drawn to receive one of 25 portable power banks (i.e., phone/tablet charger), valued at $40 each. Data were analyzed using SPSS v. 28 analytical software.

#### Results

The survey contained a list of 15 methods through which participants may have learned about SPARK, with instructions for them to select all the ways they heard about the study prior to enrolling. Table 4 shows results for participants who endorsed hearing about the study through each method, as well as the proportions who rated each method as most influential in their decisions to enroll in the study. *Social media* was the most commonly reported way people learned about the study (*n* = 1,946, 47.0%), followed by *speaking with a medical provider* (*n* = 958, 23.1%), an *online search* (which included the SPARK website and/or hospital/clinic website; *n* = 833, 20.1%), and *flyer/print material* (*n* = 529, 12.8%), while *newspaper* (*n* = 20), *radio* (*n* = 38), and *transit ads* (*n* = 14) were the least frequently endorsed (≤ 1% each). Most participants said that they heard about SPARK only one time (*n* = 1,875, 45.2%) or between two and four times (*n* = 1,801, 43.5%) prior to enrolling. Strategies that were significantly associated with return of the saliva kit for the individual with ASD included *speaking with a medical provider* (24.4% vs. 17.2%, χ^2^[1] = 16.874, *p* < .001), *speaking with a study-team member at the doctor’s office* (8.7% vs. 5.1%, χ^2^[1] = 9.967, *p* = .002), and *speaking with a study-team member at a community event* (5.9% vs. 3.8%, χ^2^[1] = 5.105, *p* = .024); hearing about the study via *social media* was associated with a significantly lower rate of saliva-kit return (45.5% vs. 54.0%, χ^2^[1] = 17.135, *p* < .001).

**Table 4.** Frequencies of participants who endorsed learning about SPARK through various recruitment outlets and ratings of those most influential in their decisions to enroll

Recruitment Strategy	Heard About SPARK This Way^a^	Most Influential in Decision to Enroll
Flyer or print material	529 (12.8%)	150 (3.6%)
Radio advertisement	38 (0.9%)	11 (0.3%)
Mailed letter	219 (5.3%)	108 (2.6%)
Friend/family member^b^	549 (13.2%)	330 (8.0%)
Transit advertisement^c^	14 (0.3%)	6 (0.1%)
Newspaper	20 (0.5%)	8 (0.2%)
Social media^d^	1,946 (47.0%)	1,270 (30.6%)
Online search^e^	833 (20.1%)	537 (13.0%)
News story^f^	140 (3.4%)	67 (1.6%)
Spoke with medical provider^g^	958 (23.1%)	733 (17.7%)
Spoke with team in clinic^h^	334 (8.1%)	215 (5.2%)
Spoke with team at event^i^	228 (5.5%)	180 (4.3%)
Newsletter/bulk email	170 (4.1%)	76 (1.8%)
Personal email from SPARK team	377 (9.1%)	235 (5.7%)
Other	295 (7.1%)	202 (4.9%)

*Note*: ^a^Specifically refers to ways heard about the study prior to enrollment. Participants were instructed to select all that apply, so proportions exceed 100%. ^b^Included any person not directly involved in the study. ^c^On a bus, train, etc. ^d^Facebook, Twitter, etc. ^e^Included the SPARK website or hospital/clinic website. ^f^Online or TV. ^g^Doctor, nurse, therapist, etc. ^h^Spoke with SPARK staff in clinic before or after doctor’s appointment. ^i^Spoke with SPARK staff at a community event (resource fairs, walks, conferences, etc.).

**Figure 1.**
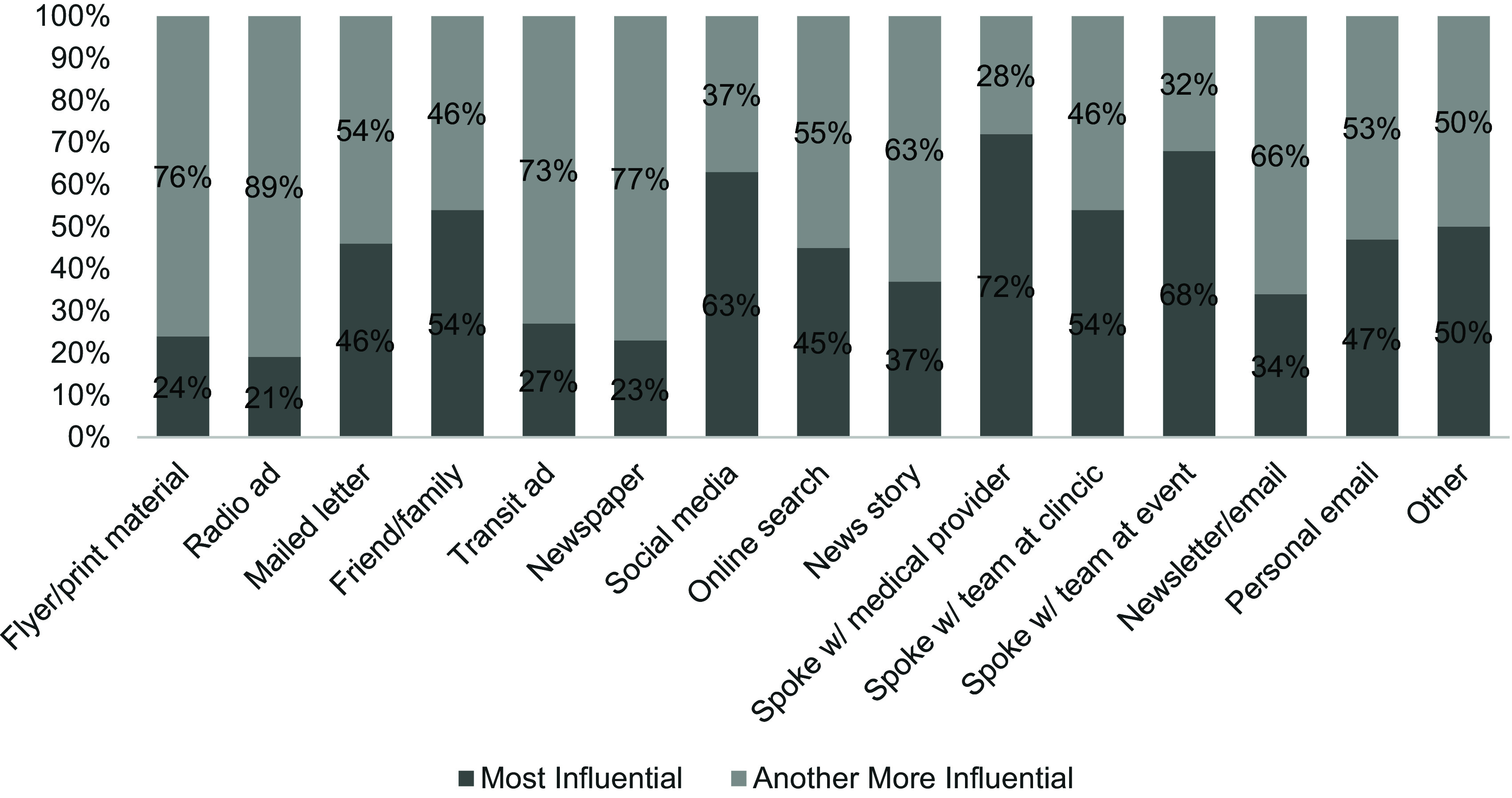
Participant ratings of most influential strategies among ways heard about SPARK*. *Note. Among participants who indicated that they learned about SPARK through a given strategy, we calculated the proportion who then rated that strategy as most influential in their decision to enroll. For example, among participants who said they learned about SPARK through a flyer/print material, 24% rated this outlet as most influential in their decision to enroll, while 76% rated an alternate outlet as most influential.

Participants who endorsed learning about SPARK from a *medical provider* rated that method as most influential in their decisions to enroll (72.1%). Other methods commonly rated as most influential were *speaking with the study team at a community event* (68.4%), *social media* (63.0%), *friend or family member* (54.8%), and *speaking with a study-team member at the doctor’s office* (54.8%). When a participant said that they had heard about SPARK through a given outlet but did not rate that outlet as most influential in their decision to enroll, the alternate strategies commonly endorsed as most influential were *social media*, an *online search*, *speaking with a medical provider*, and hearing about the study from a *friend or family member*. Additional results can be seen in Fig. 1.

With respect to demographic influences on recruitment strategies, males more often than females rated hearing about the study from a *friend or family member* (12.1% vs. 7.4%) or via an *online search* (17.1 % vs. 12.5%) as most influential in their decision to enroll, whereas females more often than males endorsed *social media* (31.5% vs. 20.8%) as most influential. Younger individuals rated hearing about the study via *social media* as most influential in their decision to enroll (42.4% of those 18–29 vs. 13.0% of those 60+), while older individuals endorsed an *online search* as most influential (16.9% of those 60+ vs. 12.3% of those 30–44). Both the youngest (age 18–29, 13.2%) and oldest (age 60+, 13.0%) individuals rated hearing about the study from a *friend or family member* as most influential in their decision to enroll compared to those in the two middle-aged groups (age 30–44, 7.2%; age 45–60, 7.6%). Differences were also observed across racial groups, as American Indian/Alaska Native participants (41.4%) and Native Hawaiian/Other Pacific Islander participants (42.9%) more often endorsed hearing about the study via *social media* as most influential in their decision to enroll, compared to Asian participants (15.2%), who least frequently indicated *social media* as most influential. Asian participants more often rated an *online search* (22.0%) and *speaking with a medical provider* (18.2%) as most influencing their decision to enroll, whereas Black/African American participants most often endorsed *social media* (30.3%), *speaking with a medical provider* (15.9%), and receiving a *personal email* from the study team (8.4%) as most influential. Hispanic/Latinx participants most often rated hearing about the study via *social media* (29.1%), their *medical provider* (17.4%), and an *online search* (15.1%) as most influential in their decision to enroll. Complete results can be seen in Tables 5 and 6.

**Table 5.** Most influential recruitment strategies by gender and age

	Gender^a^		Age (in years)			
Recruitment Strategy	Male	Female	18–29	30–44	45–60	60+
Flyer or print material	15 (3.4%)	132 (3.6%)	6 (1.6%)	93 (3.7%)	49 (4.2%)	2 (2.6%)
Radio advertisement	0 (0%)	11 (0.3%)	0 (0%)	10 (0.4%)	1 (0.1%)	(0%)
Mailed letter	8 (1.8%)	97 (2.7%)	1 (0.3%)	64 (2.5%)	40 (3.4%)	2 (2.6%)
Friend/family member^b^	53 (12.1%)	267 (7.4%)	50 (13.2%)	182 (7.2%)	88 (7.6%)	10 (13.0%)
Transit ad^c^	3 (0.7%)	2 (0.1%)	2 (0.5%)	3 (0.1%)	0 (0%)	1 (1.3%)
Newspaper	1 (0.2%)	7 (0.2%)	1 (0.3%)	5 (0.2%)	2 (0.2%)	0 (0%)
Social media^d^	91 (20.8%)	1144 (31.5%)	161 (42.4%)	825 (32.8%)	272 (23.4%)	10 (13.0%)
Online search^e^	75 (17.1%)	454 (12.5%)	50 (13.2%)	309 (12.3%)	165 (14.2%)	13 (16.9%)
News story^f^	13 (3.0%)	53 (1.5%)	2 (0.5%)	34 (1.4%)	26 (2.2%)	5 (6.5%)
Medical provider^g^	76 (17.4%)	648 (17.9%)	53 (13.9%)	473 (18.8%)	192 (16.5%)	13 (16.9%)
Team in clinic^h^	13 (3.0%)	202 (5.6%)	17 (4.5%)	151 (6.0%)	47 (4.0%)	0 (0%)
Team at event^i^	21 (4.8%)	158 (4.4%)	5 (1.3%)	115 (4.6%)	58 (5.0%)	2 (2.6%)
Newsletter/bulk email	10 (2.3%)	65 (1.8%)	6 (1.6%)	32 (1.3%)	36 (3.1%)	2 (2.6%)
Personal email^j^	30 (6.8%)	203 (5.6%)	14 (3.7%)	101 (4.0%)	111 (9.5%)	8 (10.4%)
Other	26 (5.9%)	172 (4.7%)	9 (2.4%)	115 (4.6%)	68 (5.8%)	9 (11.7%)

*Note*: ^a^20 participants reported as transgender; 13 (65.0%) rated social media as most influential in their decision to enroll. 38 participants reported as gender queer/gender nonconforming; 16 (42.1%) rated social media as most influential in their decision to enroll, while 12 (31.6%) rated friend/family member or an online search as most influential. 2 participants reported as some different gender identity; 1 (50.0%) rated social media and 1 (50.0%) rated speaking with a medical provider as most influential in their decision to enroll. 10 participants preferred not to answer about gender; 6 (60.0%) rated social media or friend/family member as most influential in their decision to enroll. ^b^Included any person not directly involved in the study. ^c^On a bus, train, etc. ^d^Facebook, Twitter, etc. ^e^Included the SPARK website or hospital/clinic website. ^f^Online or TV. ^g^Spoke with a medical provider (doctor, nurse, therapist, etc.) ^h^Spoke with SPARK staff in clinic before or after doctor’s appointment. ^i^Spoke with SPARK staff at a community event (resource fairs, walks, conferences, etc.). ^j^Personal email from a study-team member.

**Table 6.** Most influential recruitment strategies by Hispanic/Latinx ethnicity and race

	Ethnicity	Race^a^					
Recruitment Strategy	Hispanic	AS	AA	AI	PI	W	Oth
Flyer or print material	25 (5.7%)	6 (4.5%)	16 (6.4%)	2 (2.0%)	0 (0%)	118 (3.2%)	12 (7.8%)
Radio advertisement	3 (0.7%)	0 (0%)	0 (0%)	1 (1.0%)	0 (0%)	10 (0.3%)	0 (0%)
Mailed letter	9 (2.1%)	4 (3.0%)	4 (1.6%)	2 (2.0%)	0 (0%)	98 (2.7%)	5 (3.2%)
Friend/family member^b^	26 (6.0%)	6 (4.5%)	17 (6.8%)	6 (6.1%)	1 (4.8%)	299 (8.1%)	13 (8.4%)
Transit ad^c^	2 (0.5%)	0 (0%)	1 (0.4%)	0 (0%)	0 (0%)	5 (0.1%)	1 (0.6%)
Newspaper	1 (0.2%)	0 (0%)	0 (0%)	0 (0%)	0 (0%)	8 (0.2%)	0 (0%)
Social media^d^	127 (29.1%)	20 (15.2%)	76 (30.3%)	41 (41.4%)	9 (42.9%)	1157 (31.5%)	39 (25.3%)
Online search^e^	66 (15.1%)	29 (22.0%)	28 (11.2%)	16 (16.2%)	3 (14.3%)	465 (12.7%)	26 (16.9%)
News story^f^	1 (0.2%)	3 (2.3%)	6 (2.4%)	1 (1.0%)	0 (0%)	60 (1.6%)	1 (0.6%)
Medical provider^g^	76 (17.4%)	24 (18.2%)	40 (15.9%)	14 (14.1%)	4 (19.0%)	647 (17.6%)	24 (15.6%)
Team in clinic^h^	24 (5.5%)	10 (7.6%)	14 (5.6%)	4 (4.0%)	1 (4.8%)	186 (5.1%)	8 (5.2%)
Team at event^i^	19 (4.4%)	7 (5.3%)	12 (4.8%)	4 (4.0%)	1 (4.8%)	157 (4.3%)	5 (3.2%)
Newsletter/bulk email	10 (2.3%)	5 (3.8%)	6 (2.4%)	1 (1.0%)	1 (4.8%)	63 (1.7%)	4 (2.6%)
Personal email^j^	24 (5.5%)	8 (6.1%)	21 (8.4%)	3 (3.0%)	0 (0%)	199 (5.4%)	7 (4.5%)
Other	18 (4.1%)	8 (6.1%)	10 (4.0%)	3 (3.0%)	1 (4.8%)	183 (5.0%)	9 (5.8%)

*Note*: ^a^Racial categories are abbreviated as follows: AS = Asian; AA = Black/African American; AI = American Indian/Alaska Native; PI = Native Hawaiian or Other Pacific Islander; *W* = White; Oth = Other race. Participants had the option to select any/all racial categories to describe themselves. ^b^Included any person not directly involved in the study. ^c^On a bus, train, etc. ^d^Facebook, Twitter, etc. ^e^Included the SPARK website or hospital/clinic website. ^f^Online or TV. ^g^Spoke with a medical provider (doctor, nurse, therapist, etc.) ^h^Spoke with SPARK staff in clinic before or after doctor’s appointment. ^i^Spoke with SPARK staff at a community event (resource fairs, walks, conferences, etc.). ^j^Personal email from a study-team member.

Differences were also observed in how groups of primary account holders rated the influence of recruitment strategies. Compared to parents/legally authorized representatives, independent adults with autism were more likely to endorse *social media* (43.9% vs. 28.9%), hearing about the study from a *friend or family member* (15.9% vs. 6.9%), and an *online search* (19.1% vs. 12.1%) as most influential in their decisions to enroll in SPARK. Conversely, parents/legally authorized representatives were more likely than independent adults to endorse *speaking with a medical provider* (20.0% vs. 2.5%), *speaking with a study-team member at the doctor’s office* (5.9% vs. 0.6%), and *speaking with the study team at a community event* (4.8% vs. 1.2%) as most influential in their decisions to enroll. Complete results can be seen in Table 7.

**Table 7.** Most influential recruitment strategies by participant role

	Participant Role	
Recruitment Strategy	Independent Adult with Autism	Parent/LAR^a^
Flyer or print material	6 (1.2%)	144 (4.0%)
Radio advertisement	1 (0.2%)	10 (0.3%)
Mailed letter	4 (0.8%)	104 (2.9%)
Friend/family member^b^	83 (15.9%)	247 (6.9%)
Transit advertisement^c^	4 (0.8%)	2 (0.1%)
Newspaper	1 (0.2%)	7 (0.2%)
Social media^d^	229 (43.9%)	1041 (28.9%)
Online search^e^	100 (19.1%)	437 (12.1%)
News story^f^	13 (2.5%)	54 (1.5%)
Medical provider^g^	13 (2.5%)	720 (20.0%)
Team in clinic^h^	3 (0.6%)	212 (5.9%)
Team at event^i^	6 (1.2%)	174 (4.8%)
Newsletter/bulk email	6 (1.2%)	70 (1.9%)
Personal email^j^	27 (5.2%)	208 (5.8%)
Other	27 (5.2%)	175 (4.9%)

*Note*: ^a^LAR = legally authorized representative. ^b^Included any person not directly involved in the study. ^c^On a bus, train, etc. ^d^Facebook, Twitter, etc. ^e^Included the SPARK website or hospital/clinic website. ^f^Online or TV. ^g^Spoke with a medical provider (doctor, nurse, therapist, etc.) ^h^Spoke with SPARK staff in clinic before or after doctor’s appointment. ^i^Spoke with SPARK staff at a community event (resource fairs, walks, conferences, etc.). ^j^Personal email from a study-team member.

## Discussion

We assessed study personnel’s and research participants’ perspectives about effective recruitment strategies for the national SPARK study on individuals with autism and their families. Queries of study personnel focused on strategies that they had tried, perceived costs, and those they would or would not repeat/recommend, whereas queries of SPARK participants focused on the various ways they learned about SPARK and, among those, which they deemed most influential in their decisions to enroll. Study personnel reported using a wide variety of recruitment strategies, which is consistent with recommendations in the literature [e.g., 15]. Relatively large proportions cited collaborations with medical providers and paid social media as effective strategies, which have also been supported in prior reports [e.g., 14]. Yet the recruitment methods that a majority of study personnel endorsed as most effective overall (i.e., would repeat/recommend) were those that involved direct engagement with the study team (i.e., *speaking with SPARK team members in clinic*, *on-site registration/saliva-collection events*). This is consistent with findings in Denhoff et al. [19], as well as those in Daniels et al. [21] in that clinical sites had higher numbers of participants who returned saliva kits compared to other forms of recruitment, suggesting that personal connections made between study staff and potential participants can positively influence engagement and retention of families in research. Because one of the primary objectives of SPARK is to identify genes associated with autism, ensuring saliva-kit return for DNA analysis is a critical feature of the study (although not a requirement for participation).

Regarding strategies that study personnel reportedly would *not* repeat or recommend to others (i.e., *blog post*, *radio PSA*, *radio or podcast interview*), relatively few participants had tried these approaches. Although radio advertising has been used for decades, reports of its success vary; and it can be expensive [14] – a sentiment also reported by personnel in Study 1. Blogs and podcasts, however, are newer communication platforms without much evidence for or against their adoption in participant recruitment. Researchers at institutions with existing podcast stations and/or blog outlets could leverage these tools for research recruitment and evaluate their reach/impact to contribute to this knowledge base, particularly considering that study personnel rated blog posts and podcast interviews among the less costly recruitment strategies. With respect to perceptions of cost for other strategies, most were rated as “least” or “somewhat” costly. It seems likely that “return on investment” factored into study personnel’s efficacy ratings for each strategy, as some strategies that were rated “more/most costly” were not likely to be repeated/recommended (e.g., *radio advertising*) whereas others were (e.g., *on-site registration/saliva-collection events*).

Comparing results from both studies, it is interesting to note that some of the recruitment strategies that study personnel rated as most effective were those least frequently indicated by SPARK participants as ways that they learned about the study. For example, among study personnel who had used an *online news story* or a *TV news story or interview*, more than 40% were highly likely and approximately a third were somewhat likely to repeat/recommend these strategies, whereas only 3.4% of SPARK participants said they learned about the study through a news story, and more than 60% of this group rated some other strategy as more influential in their decision to enroll. Similarly, few study personnel felt that their hospital/clinic website was an effective platform for recruitment (*n* = 10, 25.0%), yet SPARK participants rated *online search* as the third most common way that they heard about the study. Interestingly, while relatively few participants learned about the study via a member of the study team in clinic (*n* = 334, 8.1%) or at an event (*n* = 228, 5.5%), 54% and 68% of them, respectively, rated those methods as most influencing their decisions to enroll. Taken together, these observations highlight the value of understanding which recruitment strategies are most likely to *reach* and *resonate* with the target audience so that limited resources can be allocated to those activities.

## Recommendations

Our findings have important implications for aiding research teams in the selection of recruitment strategies that facilitate timely enrollment of study participants. In line with suggestions to employ a variety of strategies, study personnel should consider developing recruitment plans with strategies that enhance overall awareness and engagement. For example, in the current study, 47% of SPARK participants said that they heard about the study via social media; 63% of this group said this was the most influential factor in their decision to enroll, while 11% of this group said that the online search was the most influential. This suggests that investing in paid social media that directs interested parties to a compelling website may work in concert to increase participant engagement. Similarly, 23% of SPARK participants said that they heard about the study via a medical provider; 72% of this group rated this method as the most influential in their decision to enroll, while 9% said it was speaking with a member of the study team during their clinical visit. At the same time, 13% of participants said they heard about the study via a flyer or print material; 24% of this group rated this method as most influential, whereas another 24% said speaking with a medical provider was most influential. Taken together, this suggests that (a) engaging medical providers in study referrals, (b) ensuring that families speak with a study-team member during clinical visits, and (c) providing families with study flyers during recruitment conversations with medical providers and/or study staff may collectively enhance participant engagement more so than any of these strategies alone.

The most common and influential ways that participants learned about SPARK were fairly consistent across participant groups (i.e., *speaking with a medical provider*, *social media*, or *friend/family member*). These likely represent “core” strategies that resonate with the general population, and for that reason, study teams should prioritize these strategies when outreach is broad and inclusive. Yet our results also revealed important differences in how individuals learn about and are influenced by *particular* recruitment strategies based on gender, race, ethnicity, and type of account holder (parent/legally authorized representative, independent adult with autism). Males more often reported hearing about the study through a *friend/family member*, which suggests that snowball sampling methods that encourage males to recruit other males in their social networks may effectively increase male representation in study samples. Similarly, compared to all other racial groups, Asian participants were much less likely to report hearing about SPARK through *social media* and more likely to report hearing about it via online searches. This could indicate that *paid Google advertising* or *paid website ads* – which were infrequently used by research personnel in Study 1 – could be an effective strategy to increase study visibility in a way that appeals to diverse audiences, thereby enhancing representativeness. Additionally, independent adults with autism were much more likely to hear about the study and be influenced to join via *social media* and *online searches*, whereas parents/legally authorized representatives were more likely to enroll when they heard about the study in clinic (i.e., via *medical providers*, *study teams at the doctor’s office*). This suggests that social media/online platforms as more effective in reaching adults with autism; however, it is important to note that most of the SPARK clinical sites were in pediatric departments/hospitals, so strategies that leveraged medical providers and in-clinic opportunities were targeted toward pediatric patients and their parents/guardians. It is possible that these same strategies could yield similar results when applied in adult-specialty practices and/or primary care. Taken together, the current findings provide at least some evidence-based guidance for how to tailor research-recruitment activities to efficiently meet enrollment objectives.

## Limitations

Our assessment of research personnel’s perceptions about effective recruitment strategies did not collect information about participants’ rationales for why they would or would not repeat/recommend particular outreach efforts. Although we queried their *perceptions* of cost for various recruitment strategies, this is not a metric of *actual* cost, the latter of which certainly has implications for whether and to what extent study personnel can pursue particular strategies. Likewise, we did not directly assess the efficacy of specific recruitment strategies; rather our objective was to describe and compare (1) perceived efficacy of various recruitment strategies from the perspective of research staff (Study 1) and (2) experiences of SPARK participants, who, by definition, had been successfully recruited. However, in Study 2, we did assess saliva-kit return in relation to how SPARK participants said they were recruited into the study, which provides some indication of strategies associated with participant retention/completion. Our companion paper (Daniels et al. [21]) also reported on factors associated with SPARK-study completion, one of which was affiliation with a clinical site. It follows that these participants were likely recruited into the study via strategies that clinical sites most often reported using (i.e., *speaking with SPARK team members in clinic*, *on-site registration/saliva-collection events*, *social media*, *speaking with a medical provider in clinic*, *printed materials*); however, further research should systematically evaluate rates of study enrollment/completion for these strategies to better assess their efficacy/success. Our survey of study personnel did not explicitly query use of tools within the EHR to aid recruitment efforts because, at the time of Study 1, only one site was actively piloting use of a Best Practice Advisory to aid recruitment at the point of care. Finally, study personnel’s results are based on their experiences recruiting for the SPARK study, which may not generalize to other types of research projects.

SPARK participants for Study 2 were recruited through Research Match, which is a part of SPARK that allows current participants to be notified of additional study opportunities for which they may be eligible. Those who agree to cooperate in Research Match studies are more motivated to engage in research and may not be representative of the broader SPARK-participant cohort. Likewise, given the criteria used to identify families for inclusion in Study 2, the resulting sample may differ from the overall SPARK sample, as well as that from our companion paper (Daniels et al.[21]). Although we assessed SPARK participants’ experiences with recruitment strategies by race and ethnicity, our efforts did not examine perspectives of anyone who did not speak English. At the time of data collection, SPARK was only available to individuals who were proficient in English; however, in 2022, SPARK became available in Spanish. It will be valuable to gather perceptions about effective recruitment strategies among these Spanish-speaking families in future work to inform efforts to increase diversity within research samples.

At the time of this publication, SPARK was still actively enrolling participants. The central SPARK team routinely evaluates cohort demographic data against national data to gauge representativeness. Additionally, in the last few years, diversity, equity, and inclusion (DEI) initiatives have been implemented to support recruitment/retention of underrepresented groups in the SPARK cohort (https://sparkforautism.org/portal/page/diversity-equity-inclusion-statement/).
